# Complete Genome Sequence of *Pseudomonas fluorescens* LBUM636, a Strain with Biocontrol Capabilities against Late Blight of Potato

**DOI:** 10.1128/genomeA.00446-16

**Published:** 2016-05-26

**Authors:** Christopher K. Morrison, Amy Novinscak, Vijay J. Gadkar, David L. Joly, Martin Filion

**Affiliations:** Department of Biology, Université de Moncton, Moncton, New Brunswick, Canada

## Abstract

Herein provided is the full-genome sequence of *Pseudomonas fluorescens* LBUM636. This strain is a plant growth-promoting rhizobacterium (PGPR) which produces phenazine-1-carboxylic acid, an antibiotic involved in the biocontrol of numerous plant pathogens, including late blight of potato caused by the plant pathogen *Phytophthora infestans*.

## GENOME ANNOUNCEMENT

*Pseudomonas fluorescens* strain LBUM636 is a Gram-negative, rod-shaped bacterium isolated from agricultural soil in New Brunswick, Canada. This strain displays broad antagonistic activity against many plant pathogens (1). Preliminary results have shown that LBUM636 is capable of biocontrol activity against late blight of potato, a disease caused by the oomycete *Phytophthora infestans* (Morrison et al., unpublished data). Phenazine-1-carboxylic acid (PCA) production by *P. fluorescens* LBUM636 was shown to be essential for its biocontrol capabilities. PCA production has previously been linked to disease suppression in different pathosystems (2, 3). The strain also displays potato plant growth-promoting activity (Morrison et al., unpublished data).

Genomic DNA extraction was performed using the Ultra-Clean microbial DNA isolation kit (Mo Bio, Carlsbad, CA, USA) and the DNA was purified using the Agencourt AMPure XP purification kit (Beckman Coulter, Mississauga, Canada). The genome of LBUM636 was sequenced at the McGill University and Génome Québec Innovation Centre (Montreal, Quebec, Canada) using the next-generation Pacific Biosciences single-molecule real-time sequencing technology on a PacBio RSII platform. It generated a total number of 207,124 raw subreads with an average length of 5,795 bp (156× coverage). The assembly of the genome was performed using the Hierarchical Genome Assembly Process (HGAP) (4), and generated a circular chromosome of 6,868,221 bp in length with no functional plasmid being detected. G+C content was determined at 60.55%. Genome annotation with the RAST server (5) predicted the presence of 6,183 protein-coding sequences, 65 tRNA genes, and 5 rRNA operons.

Ten core housekeeping genes *(acsA*, *aroE*, *dnaE*, *guaA*, *gyrB*, *mutL*, *ppsA*, *pyrC*, *recA* and *rpoB* [6]) were retrieved from GenBank for 48 closely related *Pseudomonas* spp. and compared by concatenated alignments using the CLC Genomics Workbench software version 8.0 (CLC bio, Boston, MA, USA). The maximum-likelihood phylogenetic tree generated by the software indicates that *P. fluorescens* LBUM636 belongs to *P. fluorescens* subclade 3 according to Loper et al. (6). *P. fluorescens* LBUM636’s closest identified relative is *P. fluorescens* UK4 (accession no. CP008896). Analysis of the genome indicated the presence of a single copy of the *phzABCDEFG* operon necessary for the production of PCA, a feature not found in subclade 3 isolates described in Loper et al. (6) but found in another *P. fluorescens* strain previously sequenced by our group (7). Also found were genes potentially associated with plant growth-promotion activity such as genes involved in the production of 1-aminocyclopropane-1-carboxylate deaminase (*acdS*) and pyrroloquinoline-quinone (*pqqABCDEF*). Genes involved in the production and the uptake of the siderophore pyoverdine were also found within the genome.

### Nucleotide sequence accession numbers.

This complete genome project has been deposited in DDBJ/ENA/GenBank under the accession no. CP012400. The version described in this paper is the first version, CP012400.1.
